# Successful Antimicrobial Treatment of Phlegmonous Gastritis: A Case Report and Literature Review

**DOI:** 10.1155/2018/8274732

**Published:** 2018-09-16

**Authors:** Madiha Iqbal, Rabia Saleem, Salman Ahmed, Prachi Jani, Salvador Alvarez, Han W. Tun

**Affiliations:** ^1^Department of Hematology and Oncology, Mayo Clinic, Jacksonville, FL, USA; ^2^University of Oklahoma, Oklahoma City, OK, USA

## Abstract

Phlegmonous gastritis is an uncommon acute bacterial infection of the stomach that carries a fatal prognosis in spite of the advent of antibiotics. A high index of suspicion is required in patients with risk factors. An immunocompromised state is identified as one of the most important risk factors. We hereby report a case of successful antimicrobial treatment of phlegmonous gastritis in a patient who was receiving intensive chemotherapy for acute myelogenous leukemia. We have also carried out a review of literature over the past ten years. *Streptococcus pyogenes* is identified as the most common causative organism, and patient presentation is usually nonspecific. Conservative treatment with prompt institution of antibiotics can lead to rapid resolution in the majority of patients.

## 1. Introduction

Phlegmonous gastritis (PG) is a rare acute bacterial infection, which primarily involves the submucosal layer of the stomach wall, but can also involve the muscularis layer and rarely the mucosa [1–3]. It is rapidly fatal if untreated and thus requires prompt diagnosis and management. Even with the correct diagnosis and antimicrobial therapy, the mortality rate remains high at 27–40% [2, 3]. PG primarily affects the middle-aged population of 45–74 years, with a 65% male predominance [4]. The underlying etiology is largely unknown, although an immunocompromised state associated with malignancy, chemotherapy-induced neutropenia, acquired immunodeficiency syndrome (AIDS), alcoholism, and immunosuppressive drugs is considered an important risk factor [5]. We herein report a patient who developed PG in the setting of prolonged neutropenia related to relapsed acute myeloid leukemia (AML) and intensive chemotherapy and was successfully treated with systemic antimicrobial therapy.

## 2. Case Presentation

**Figure 1:**
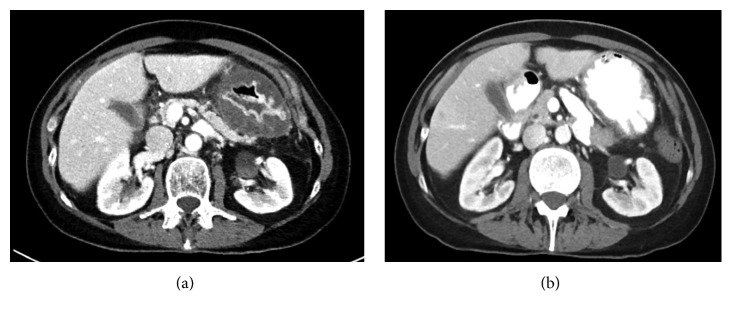
(a) Diffuse thickening of the stomach wall with mucosal hyperenhancement and marked submucosal edema. (b) Marked interval improvement in gastric wall thickening and greater curvature intramural hypodensity after two weeks of antibiotic treatment.

The patient reported is a 56-year-old woman who was diagnosed with acute myeloid leukemia (AML) with cytogenetic abnormality of inversion 16 in 2013. She achieved a complete remission (CR) after standard induction chemotherapy with 7 + 3 regimen consisting of ara-C and daunorubicin followed by consolidation with high-dose ara-C (HiDAC). She relapsed a year later and was re-induced with a salvage chemotherapy regimen MEC (mitoxantrone, etoposide, and cytarabine) achieving a second CR, which was followed by a matched unrelated allogeneic stem cell transplant (allo-SCT). Her posttransplant course was uneventful without significant graft versus host disease and prolonged requirement for immunosuppression. Two years after allo-SCT, she had a central nervous system (CNS) relapse of her original leukemia and presented with an infiltrating lesion in the lumbosacral spine; her CSF cytology was positive for myeloblasts. She was admitted to the hospital to receive reinduction chemotherapy; her vitals upon admission were as follows: temperature 37.7 °C, blood pressure (BP) 129/65 mmHg, heart rate (HR) 72/min, and respiratory rate (RR) 14/min. She was started on intrathecal chemotherapy with ara-C and systemic chemotherapy with the salvage chemotherapy regimen FLAG-IDA (fludarabine, ara-C, and idarubicin). The day chemotherapy started for the patient was noted as day 1. On day 10, the patient developed neutropenic fever, and the white blood count (WBC) noted to be <0.1 × 10^9^/L with absolute neutrophil count (ANC) of 0. She was started on intravenous (IV) cefepime 2 g every 8 hour after evaluation for underlying infectious etiology was done. The work up did not isolate any organism and included blood culture, urine culture, and chest X-ray. On day 16, the patient developed left upper quadrant abdominal pain. Vital signs then were as follows: maximum temperature (*T*_max_) 37.5°C, along with HR of 80–94/min, RR 16–18/min, and BP SBP 105–126/DBP 55–71 mmHg. Her blood tests then were as follows: WBC <0.1 × 10^9^/L, ANC 0, hemoglobin 8.0 g/dl, platelet 12 × 10^9^/L, and serum blood chemistry and liver function tests were noted to be without significant derangements. A CT scan of the abdomen was performed that showed diffuse thickening of stomach wall (Figure 1(a)), concerning for infectious or infiltrative malignant process. Her absolute neutrophil count had been at the nadir for 10 days prior to this development. Her antimicrobial coverage was increased to include anaerobic coverage by changing her antibiotic regimen from IV cefepime 2 g every 8 hour to IV piperacillin/tazobactam 3.375 g every 6 hour, leading to short-lived symptomatic improvement for roughly two weeks. Upon symptom recurrence, the patient was noted to be febrile with *T*_max_ of 39.5°C, along with HR of 109–139/min, RR 18–20/min, and BP SBP 94–124/DBP 55–71. Two sets of peripheral blood cultures were drawn which did not show growth of any organism after 5 days of incubation. The patient continued to remain hemodynamically stable.

**Figure 2:**
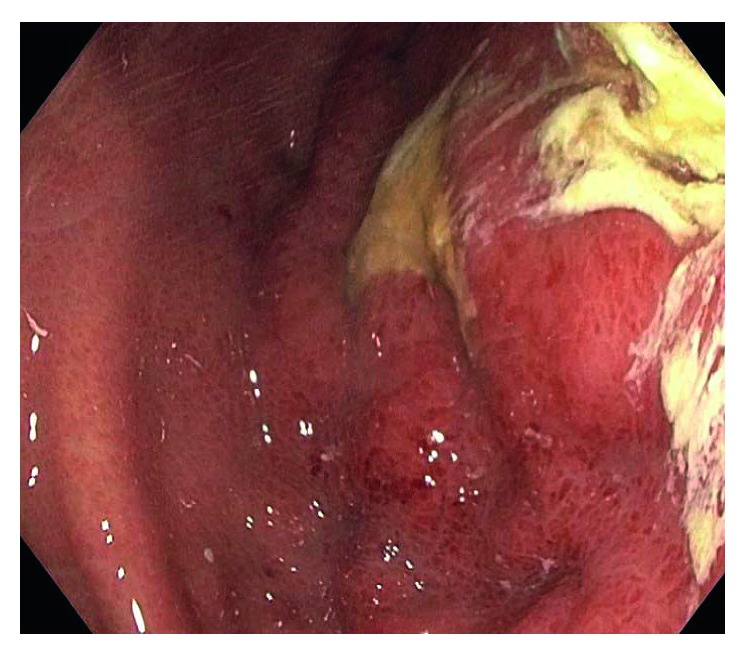
Upper endoscopy showing large nonobstructing nonbleeding gastric deep ulcerations with exudate material.

An upper gastrointestinal (GI) endoscopy was performed which showed a large ulcerative lesion with purulent discharge and inflammatory changes (Figure 2). *Citrobacter freundii*, *Enterococcus faecalis*, and *Bacillus cereus* were isolated from culture on gastric biopsies. Imaging, endoscopic, and microbiological findings were consistent with phlegmonous gastritis.

Infectious disease service was consulted, IV piperacillin/tazobactam 3.375 g every 6 hour was stopped, and the antibiotic regimen was changed to IV vancomycin (managed per pharmacy protocol based on weight and renal function) and IV meropenem 1 g IV every 8 hour. The recommendation for broad coverage of microorganisms was made by infectious disease service, given the high risk of mortality associated with phlegmonous gastritis. Prophylactic antifungal and antiviral for neutropenia were continued. Organism susceptibilities were carried out for *Citrobacter freundii* and *Enterococcus faecalis*, which revealed *Citrobacter freundii* to be resistant to ampicillin, cefazolin, and cefuroxime, while *Enterococcus faecalis* was noted to be pansensitive. Per organism susceptibility and with the help of infectious disease service, her antibiotic regimen was changed to IV cefepime 2 g every 8 hour, IV metronidazole 500 mg IV every 8 hour, and IV vancomycin per pharmacy protocol. This antibiotic regimen was continued for a total of two weeks. The patient's gastrointestinal symptoms resolved quickly, and she was able to resume normal diet. Follow-up CT scan a month later showed marked improvement in gastric thickening (Figure 1(b)). She is currently doing well with her AML in remission and no recurrence of her GI symptoms.

## 3. Discussion and Literature Review

Phlegmonous gastritis is a rare infection with less than 500 cases reported in literature [3]. We have carried out a bibliographical search using PubMed from 2007 to 2017 using the keyword “Phlegmonous Gastritis” and found 36 articles, in English, Spanish, and Japanese, of which we reviewed 25.

Our limited data review showed that there have been only 4 cases reported in the USA and 3 in Europe in the last 10 years, whereas the bulk was reported in Southeast Asia, namely Japan (13) and Korea (8). In addition to an immunocompromised state, the other reported risk factors are increased age, gastric mucosal injury from chronic gastritis, peptic ulcer, endoscopic procedures, gastric cancer, achlorhydria, infection, and malnutrition [6, 7]. Our review found malignancy to be a risk factor in 32% of the cases (8/25). The mortality rate has been reported to be higher in groups with an identified risk factor as compared to those without one [8]. PG has been further classified into primary and secondary types [1]. The primary type is usually idiopathic or occurs after direct damage to the gastric mucosa due to trauma, cancer, and endoscopic interventions, thereby leading to direct microbial invasion. The secondary type is either associated with infection of neighboring organs, such as infection due to pancreatitis, hepatic abscess, and cholecystitis, or hematogenous/lymphogenous spread from other organs.

PG is a rare infection that usually presents with nonspecific gastrointestinal symptoms. A high index of suspicion is necessary, especially in immunocompromised individuals. Epigastric/abdominal pain is the most common symptom, with other symptoms including but not limited to being fever, nausea, vomiting, and, less often, diarrhea and hematemesis [6]. PG should be highly suspected if a CT scan shows diffuse thickening of the stomach wall. The upper GI endoscopy should be performed as visual and microbiologic findings help establish the diagnosis and guide antimicrobial therapy. Although, *Streptococcus pyogenes* is the most frequently reported isolated organism in about 70% of cases, polymicrobial infection as seen in our patient is also quite common [2, 8, 9]. Our review of cases followed a similar pattern, wherein *Streptococcus* spp. was the most common pathogen identified in 44% of cases, but there were also other uncommon pathogens isolated such as *Citrobacter*, *Acinetobacter*, *Enterobacter* spp., and *Bacillus* spp., and even resistant pathogens like MDR (multidrug-resistant) *Streptococcus* and VRE (vancomycin-resistant enterococcus) (Table 1).

It is imperative to initiate empiric broad-spectrum antimicrobial coverage as soon as there is the clinical suspicion of possible phlegmonous gastritis, followed by adjustments as per microbiological culture results and clinical course. Early diagnosis and prompt institution of antibiotics has been shown to prevent patient mortality and defer the need of a surgical intervention [7]. All of the patients in our review received antibiotics, with resolution seen in 22/25 (88%) of the cases and death in 3/25 of the cases (12% mortality). This illustrates that prompt recognition and awareness of phlegmonous gastritis as possible complication of prolonged neutropenia in patients with hematological malignancies can clearly impact outcomes.

Invasive modality for management includes surgical gastrectomy and is mainly reserved for those with impending local complications such as perforation or sepsis [6, 7]. As mentioned above if promptly recognized, majority of the patients can be treated via conservative measures. Patients with hematologic malignancies are a unique population that is extremely susceptible to this complication due to prolonged neutropenia, secondary to intensive chemotherapy regimens. Prompt intervention and assistance from multiple medical specialties is needed to prevent fatality from this aggressive infection. Our case highlights the need for awareness of this rare infectious complication amongst health care professionals who take care of immunocompromised patients especially those suffering from hematological malignancies as knowledge of this rare complication can clearly impact patient mortality. Table 1 shows the major clinical characteristics, imaging findings, microbiology results, treatment, and survival outcomes from our literature review.

## Figures and Tables

**Table 1:** Pertinent findings from our review of 25 case reports.

Author/Year	Sex	Age	Cause/risk factor	Symptoms	Pathogen/s	Diagnosis	Intervention/management	Complications	Result
Ajibe H, 2008	M	74	Endoscopic submucosal dissection for gastric adenocarcinoma	Epigastric pain, fever	*Citrobacter freundii*, *Enterobacter cloacae*, *and Streptococcus*	CT, EGD, EUS	Antibiotics, total gastrectomy	Nil	Discharge
Alonso et al., 2013 [7]	F	55	None	Chest pain	*Streptococcus pyogenes*	CT	Antibiotics, endoscopic abscess drainage	Nil	Discharge
Fan JQ, 2013	M	65	Esophagectomy for esophageal adenocarcinoma	Fever, dyspnea	Nil	CT, EGD	Antibiotics	Nil	Discharge
Flor-de‐Lima F, 2015	M	7	Acute tonsillitis	Epigastric pain, nausea, vomiting	*Streptococcus pneumoniae*, EBV	CT, EGD with biopsy	Antibiotics	Nil	Discharge
Guo et al., 2009 [3]	M	57	CML, myeloid sarcoma	Dyspnea, chest pain, abdominal pain, fatigue	VRE	CT, EGD with biopsy, autopsy	Antibiotics	Upper GI bleed	Death
Huang et al., 2017 [5]	F	60	Uncontrolled DM	Fever, fatigue, chest pain	*Pseudomonas*, *Klebsiella*	CT, EGD	Antibiotics, surgery (not gastrectomy)	Hypopharyngeal abscess, esophageal perforation	Discharge
Ishigami T, 2008	M	70	None	Fever, abdominal pain	MDR *Streptococcus*	EGD	Antibiotics	Nil	Discharge
Itonaga M, 2012	F	70	EUS-FNA for pancreatic tumor	Fever, abdominal pain	Alpha-hemolytic *Streptococcus*	Ct, EGD, EUS	Antibiotics	Nil	Discharge
Kato et al., 2015 [1]	M	64	DM, chronic pancreatitis	Epigastric pain, nausea	*Peptostreptococcus*	CT, EGD with biopsy	Antibiotics, EUS-guided pseudocyst drainage	Nil	Discharge
Kim BY, 2017	M	51	Ankylosing spondylitis	Nausea, vomiting	Nil	CT, EGD	Antibiotics	Nil	Discharge
Kim et al., 2010 [9]	M	48	Alcoholism, uncontrolled DM	Fever, chest pain, abdominal pain, dyspnea	*Klebsiella* spp.	EGD	Antibiotics, surgery (not gastrectomy)	Bilateral pleural effusions	Discharge
Kim et al., 2016 [2]	M	74	Alcoholic cirrhosis, DM, recent EGD, gastric adenocarcinoma	Abdominal pain, vomiting	Nil	CT, EGD with biopsy	Antibiotics	Nil	Discharge
Kim NY, 2011	M	66	None	Epigastric pain, nausea, vomiting	*Klebsiella*, *Acinetobacter*	CT, EGD with biopsy	Antibiotics	Nil	Discharge
Liu YJ, 2013	M	84	Colon cancer, prostate cancer	Fever, nausea, vomiting	Polymicrobial (gram positive and gram negative organisms)	CT, exploratory laparotomy gastric biopsy	Antibiotics, gastrectomy	Nil	Discharge
Matsumoto H, 2015	M	74	Myelofibrosis, multiple myeloma, neutropenia	Epigastric pain, nausea	*Bacillus* spp.	CT, EGD	Antibiotics	Sepsis, disseminated intravascular coagulation	Death
Min et al., 2014 [6]	F	51	Gastric ulcer	Abdominal pain	*Streptococcus pyogenes*	CT, exploratory laparotomy	Antibiotics, total gastrectomy	Gastric submucosal dissection	Discharge
Morimoto et al., 2014 [4]	M	77	DM, gastric ulcers	Fever, nausea, vomiting	Group A *Streptococcus*	CT	Antibiotics		Death
Nomura K, 2015	F	80	None	Epigastric pain, vomiting	*Enterobacter cloacae and Enterococcus faecium*	CT, EGD with biopsy	Antibiotics, total gastrectomy (worsening gastric strictures)	Nil	Discharge
Paik DC, 2010	M	45	Recent paranasal sinus surgery	Abdominal pain, nausea, vomiting	*Streptococcus pyogenes*	CT, exploratory laparotomy EGD	Antibiotics	Respiratory failure, renal failure, coagulopathy	Discharge
Park CW, 2010	F	73	Gastric outlet narrowing	Epigastric pain, abdominal distension	*E. coli*, *Acinetobacter*	CT, EGD with biopsy	Antibiotics, pyloric stent	Nil	Discharge
Rada-Palomino et al., 2014 [8]	M	62	HIV	Epigastric pain, hematemesis, diarrhea	*Streptococcus pyogenes*	CT, EGD with biopsy	Antibiotics	Nil	Discharge
Saito M, 2012	F	55	ALL	Neutropenia	*Bacillus* spp.	CT, EGD with biopsy	Antibiotics	Nil	Discharge
Sakata T, 2011	F	63	None	Fever	None reported	CT, EGD with biopsy	Antibiotics, drainage	Nil	Discharge
Shiozawa K, 2009	M	62	Uncontrolled DM	Epigastric pain	None reported	CT	Antibiotics, drainage	Nil	Discharge
Munroe CA, 2010	M	58	Chronic hepatitis B	Epigastric pain, nausea, fever	Alpha-hemolytic *Streptococcus*	CT, EGD, EUS with biopsy	Antibiotics, aspiration	Nil	Discharge

CML: chronic myeloid leukemia; DM: diabetes mellitus; HIV: human immunodeficiency virus; ALL: acute lymphoblastic leukemia; EBV: Epstein-Barr virus, VRE: vancomycin-resistant *Enterococcus*; MDR: multidrug resistance; Strep: *Streptococcus*; EUS: endoscopic ultrasound; CT: computed tomography; EGD: esophagogastroduodenoscopy; FNA: fine needle aspiration.
